# P-496. Trajectory of Veterans Aging Cohort Study Index Score in People Living with HIV

**DOI:** 10.1093/ofid/ofae631.695

**Published:** 2025-01-29

**Authors:** Lingshan Syue, Wen-Chien Ko, Nai-Ying Ko, Chung-Yi Li

**Affiliations:** National Cheng Kung University Hospital, SFO, California; National Cheng Kung University Hospital, SFO, California; National Cheng Kung University, Tainan, Tainan, Taiwan; National Cheng Kung University, Tainan, Tainan, Taiwan

## Abstract

**Background:**

HIV infection weakens human immunity and also causes chronic inflammation. Effective antiretroviral therapy (ART) restores cellular immunity but its ability to reverse chronic inflammation is limited. The Veterans Aging Cohort Study (VACS) is a longitudinal, prospective observational cohort study and used routinely obtained generic data during clinic follow to generate the VACS index which was correlated with poor outcomes. The present study aims to describe the long-term VACS index trajectory pattern of PLWH and to identify the possible factors associated with the unfavorable trajectory of PLWH.

Trajectories of the VACS index and the confidence intervals over up to 7 years of follow-up (n=1677)

**Figure:**
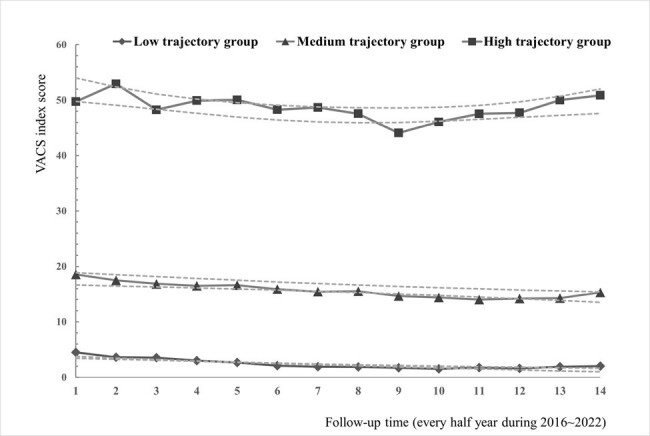

**Methods:**

This was a retrospective cohort study enrolling adult PLWH who received HIV care regularly for more than one year in a single medical center and data were retrieved from the electronic medical system. The VACS index for each patient every half year was calculated during 2016∼2022 and collected for group-based trajectory analysis (GBTA). All these analyses were done using the SAS software (9.4 version, SAS Institute Inc., Cary, NC, USA).

Multivariate odds ratio with 95% confidence intervals of factors associated with unfavorable trajectory in PLWH by the logistic regression method (n=1677)

**Figure:**
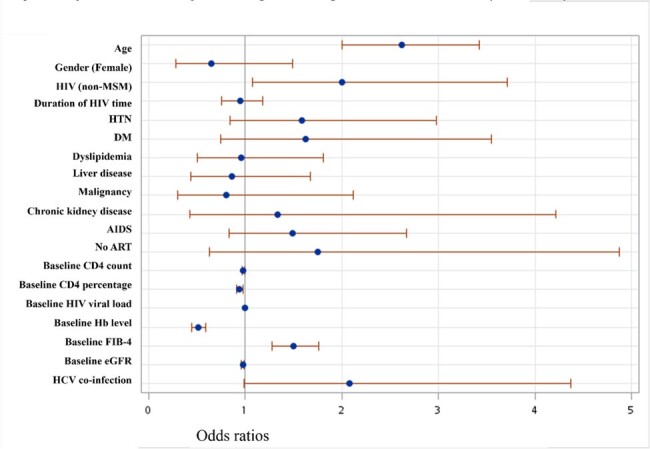

**Results:**

A total of 1677 PLWH with 13366 VACS index records were analyzed through GBTA and categorized into three trajectories: consistently low VACS index with a median VACS index score of 0 points (40.4%), consistently medium scores (median score, 16) of the VACS index (48.2%), and consistently high scores (median score, 52 ) of the VACS index (11.4%) The medium score distribution of the VACS index in the three groups remained unchanged even though the proportion of ART prescriptions increased during the entire follow-up. Age, HIV exposure other than male-to-male sexual contact, baseline CD4 count, Hb level, renal function, FIB-4, and HCV co-infection were significantly associated with the unfavorable trajectory in the multivariate model.

**Conclusion:**

The present study showed that the VACS index is a relatively stable indicator and could be used as an outcome indicator and also provided physicians with an understanding of the long-term trajectories of the VACS index in PLWH. Physicians could identify the high-risk PLWH quickly by using the VACS index. For the PLWH with the consistently high VACS index, physicians need to consider more evaluations or interventions to assure drug adherence to address HIV control.

**Disclosures:**

**All Authors**: No reported disclosures

